# Does musical enrichment enhance the neural coding of syllables? Neuroscientific interventions and the importance of behavioral data

**DOI:** 10.3389/fnhum.2014.00964

**Published:** 2014-12-16

**Authors:** Samuel Evans, Sophie Meekings, Helen E. Nuttall, Kyle M. Jasmin, Dana Boebinger, Patti Adank, Sophie K. Scott

**Affiliations:** ^1^Institute of Cognitive Neuroscience, University College LondonLondon, UK; ^2^Speech, Hearing and Phonetic Sciences, University College LondonLondon, UK

**Keywords:** musicianship, speech perception, literacy, intervention, auditory brainstem response (ABR)

Speech perception problems lead to many different forms of communication difficulties, and remediation for these problems remains of critical interest. A recent study by Kraus et al. (2014b) published in the Journal of Neuroscience, used a randomized controlled trial (RCT) approach to identify how low intensity community-based musical enrichment for “at-risk children” improved neural discrimination of “ba/ga” syllables. In the study, forty-four children aged six to nine years from “gang reduction zones” received 2 hours of musical training each week arranged in two 1 hour sessions. A control group received a single year of training following a one year delay, whilst the experimental group received two full years of training without delay. They found that auditory brainstem responses (ABRs) to the “ba/ga” syllables were changed in the experimental group, but only after more than one year of training. ABRs were not changed in the control group, either following the delay or after the first full year of training. We endorse the use of a randomized control trial (RCT) to evaluate this educational programme, but argue that several additional criteria must be met before firm conclusions can be drawn about the benefits of the intervention.

Kraus et al. argue their results provide evidence that “community music programs may stave off certain language-based challenges” (Kraus et al., 2014b, p. 11915), but this claim is hard to sustain without behavioral data (e.g., of concomitant improvements in speech perception or literacy). For the current paper, it would be necessary to show group differences in behavior that relate to the educational program, and explore the ways that individual differences in neural and behavioral profiles vary with the speech and literacy measures. This is particularly important given that a meaningful musicianship advantage in speech perception can be hard to demonstrate, as the size of the advantage shown for musicians (compared to non-musicians) is small (<1 dB) (Parbery-Clark et al., 2009) and has not been consistently replicated (Fuller et al., 2014; Ruggles et al., 2014). We also note a more recent follow up study (Kraus et al., 2014a) shows no improvement in literacy skills associated with active musical engagement.

There are other important issues: for example, Kraus et al. presented a single pair of synthesized “ba” and “ga” syllables 6000 times, at a rate of 4.35 repetitions per second, to each participant. No naturally occurring human speech sequences occur like this: speech tokens are never identical, and repetition itself is normally avoided as it is low in informational value (change, not repetition, conveys information) and leads to illusory percepts (cf. the verbal transformation effect, Pitt and Shoaf, 2002).

In addition, these items were synthesized speech tokens in which a single acoustic cue (the trajectory of the second format, F2) was manipulated. Notably, the major frequency difference where the F2s are maximally different between ba and ga (900–2480 Hz) are not investigated as the cross-phaseogram measurements are restricted to 900–1500 Hz, due to a lack of phase locking above 1500 Hz (Aiken and Picton, 2008). This frequency “window” restricts the analysis to a range where the whole F2 sweep for “ba” is included, but most of that for “ga” is excluded from the analysis (see Figure in Supplementary Materials, Hornickel et al., 2009). This suggests that the response is not specifically discriminant *per se*, and may be associated with detection of the presence of “ba” stimuli. A contrast of “ba” with “da,” which has a lower F2 sweep, would be a way to address this. To further develop our understanding of these ABR effects, it is also essential to understand how the measurements used in this study relate to the auditory brain stem and cortex measures used in other investigations, of the effects of musical training. Table 1 shows a summary of the ABR papers on musical training in children which illustrates the wide variety of measures used and their significance across studies.

**Table 1:** **A summary of the ABR papers on musical training in children which illustrates the wide variety of measures used and their significance across studies**.

**Paper**	**Age range**	**Stimulus**	**Musical training criteria**	**Measure**	**Finding (relative to non-musician/control group)**
Strait et al. (2012; Brain and Language)	School age (7–13 years)	Synthetic 170 ms /da/ stimulus presented with and without multi-talker babble noise	Currently undergoing private instrumental training, began musical training by age 5 and had practiced ≥20 min at least 5 days weekly for last 4 years	**cABR peak timing**	First peak at the start of the formant transition (43 ms) faster in quiet and in noise. No significant differences between onset peak (9 ms) or steady-state vowel peak (63 ms) in quiet or noise
					Less of a quiet-noise timing shift in formant peak (43 ms), but not in onset (9 ms) or steady-state (63 ms)
				**Fast Fourier Transform**	Stronger encoding of summed frequencies across range of 200–800 Hz in quiet and noise conditions. No difference in strength of fundamental frequency encoding in quiet or noise
				**Stimulus-response correlation**	Significant difference in strength of stimulus-response correlation in noise for vowel region. No significant difference in quiet. Significant difference in quiet vs. noise stimulus-response correlation difference
Strait et al. (2013; Developmental Cognitive Neuroscience)	Preschoolers (3–5 years)	Synthetic 170 ms /da/ stimulus presented with and without multi-talker babble noise	Currently undergoing private or group music training for minimum of 12 consecutive months before the study. Attending weekly classes and used materials to practice 4 times a week at home	**cABR peak timing**	Onset peak (9 ms) and formant transition (43 ms) faster in quiet and in noise. No significant difference in steady-state peak (63 ms) in quiet or noise.
					Less of a quiet-to-noise timing shift for formant transition peak (43 ms), but no significant differences in quiet-noise timing shifts for onset (9 ms) or steady-state vowel (63 ms) peaks
				cABR peak amplitude	No absolute amplitude differences in quiet or noise conditions, nor a difference in quiet-noise amplitude reductions for onset (9 ms), formant (43 ms) or steady-state (63 ms) peaks
				Stimulus-response correlation	No differences in stimulus-response correlation strength across vowel region in quiet or noise. No significant difference in quiet vs. noise stimulus-response correlation difference
				Fast Fourier Transform	No differences in strength of encoding at fundamental frequency or for frequencies summed across 200–800 Hz in either quiet or noise conditions
Strait et al. (2014; Cerebral Cortex)	Preschoolers (3–5 years) and school age (7–13 years)	170 ms synthetic /ba/ and /ga/ stimuli	Preschoolers: Currently undergoing private or group music training for minimum of 12 consecutive months before the study. Attending weekly classes and used materials to practice 4 times a week at home. School age: Currently receiving private lessons, started music training by or before age 6, and had consistently practiced for a minimum of 3 years for ≥20 min for at least 5 days weekly	Cross-phaseogram^*^	Better phase differentiation between /ba/ and /ga/ stimuli from 15 to 45 ms post-stimulus onset, (corresponding to formant transition) across frequency range 900–1250 Hz in preschoolers and to 900–1500 Hz in school-aged children. No phase differences in control vowel region (60–170 ms)
Kraus et al. (2014a; Frontiers in Neuroscience)	School-age (7–10 years)	40 ms consonant-formant transition /d/ (perceived as /da/)	Harmony Project music appreciation: 1 h twice per week pitch, rhythm, vocal performance, improvisation, composition, musical styles and notation, basic recorder training. Some subjects progressed to 2 h/week of other instrumental training, ensemble practice and performance	**Peak latencies, VA slope (stop burst),**	Earlier latencies for peaks V (onset), E, and F (consonant transition period) in second year of training relative to group with no instrumental training. No significant differences in latencies of peaks A, C, D, O, or slope of VA complex (onset peak-trough)
				Fast Fourier Transform	No significant differences between summed energy across (“middle harmonics”) 455–720 Hz, or across 720–1154 Hz (“high harmonics”)
Strait et al. (2011; Behavioral and Brain Functions)	School-age (8–13 years) classified as “good” and “poor” readers	Repeated /da/ (predictable context) vs. standard /da/ interspersed with /ba/, /ga/, /du/, /ta/, shorter /da/, higher-pitched /da/, dipping pitch /da/ (variable context)	This was not a training study.	**Fast Fourier Transform**	Stronger frequency encoding of 200 and 400 Hz frequency components in predictable vs. variable stimulus contexts in good readers relative to poor readers. Reading ability and music aptitude scores correlated with strength of encoding at both 200 and 400 Hz. No significant differences in strength of fundamental frequency encoding or at any other harmonic frequencies
			Measured at only one time point, music aptitude was an aggregate score that measured the ability to compare melodies and rhythms		

*Bold indicates a significant difference between the musicians and non-musicians/control group in at least one measure*.

* *Indicates the same ABR measurement used in Kraus et al. (2014b)*.

RCTs involve certain design features, which Kraus et al. do not always fully exploit: for example, the difference in the size between the control (*n* = 18) and the experimental (*n* = 26) groups is unexplained, and may require a different statistical approach (Keselman and Keselman, 1990). The lack of an active control group prevents us from understanding whether the reported neural changes could be induced by an alternative enrichment activity (which is acknowledged by the authors), or whether a more focused language or literacy intervention would have yielded more effective results. It is also important to stress that while the paper makes specific claims about treatment effects for “impoverished brains” (e.g., individuals from low socio-economic backgrounds), no direct evidence of this impoverishment is provided, nor evidence that the effects on “impoverished” brains are any different to the effects on non-impoverished brains, e.g., by including another control group. RCT methodology requires the reporting of the system used to generate the random allocation sequence, as well as mentioning participant drop-out rates, means, SDs, effect sizes and associated confidence intervals. Although an important first step, this paper falls some way short of suggested recommendations for the reporting of RCTs (Schulz et al., 2010).

To conclude, we have critiqued a recent high impact intervention study examining the effect of musical training on neural responses. Ineffective interventions provide false hope and waste financial resources (Strong et al., 2011) and therefore intervention programmes need to be evaluated rigorously. It is admirable to investigate the potential of community based musical training to improve neural coding of speech, but we argue that a stronger standard of evidence is required before concluding that musical enrichment enhances speech, language and literacy skills.

## Conflict of interest statement

The authors declare that the research was conducted in the absence of any commercial or financial relationships that could be construed as a potential conflict of interest.
